# Progress towards the 2020 targets for HIV diagnosis and antiretroviral treatment in South Africa

**DOI:** 10.4102/sajhivmed.v18i1.694

**Published:** 2017-07-27

**Authors:** Leigh F. Johnson, Rob E. Dorrington, Haroon Moolla

**Affiliations:** 1 Centre for Infectious Disease Epidemiology and Research, University of Cape Town, South Africa; 2 Centre for Actuarial Research, University of Cape Town, South Africa

## Abstract

**Background:**

The UNAIDS targets for 2020 are to achieve a 90% rate of diagnosis in HIV-positive individuals, to provide antiretroviral treatment (ART) to 90% of HIV-diagnosed individuals and to achieve virological suppression in 90% of ART patients.

**Objectives:**

To assess South Africa’s progress towards the 2020 targets and variations in performance by province.

**Methods:**

A mathematical model was fitted to HIV data for each of South Africa’s provinces, and for the country as a whole. Numbers of HIV tests performed in each province were estimated from routine data over the 2002–2015 period, and numbers of patients receiving ART in each province were estimated by fitting models to reported public and private ART enrolment statistics.

**Results:**

By the middle of 2015, 85.5% (95% CI: 84.5% – 86.5%) of HIV-positive South African adults had been diagnosed, with little variation between provinces. However, only 56.9% (95% CI: 55.3% – 58.7%) of HIV-diagnosed adults were on ART, with this proportion varying between 50.8% in North West and 72.7% in Northern Cape. In addition, 78.4% of adults on ART were virally suppressed, with rates ranging from 69.7% in Limpopo to 85.9% in Western Cape. Overall, 3.39 million (95% CI: 3.26–3.52 million) South Africans were on ART by mid-2015, equivalent to 48.6% (95% CI: 46.0% – 51.2%) of the HIV-positive population. ART coverage varied between 43.0% in Gauteng and 63.0% in Northern Cape.

**Conclusion:**

Although South Africa is well on its way to reaching the 90% HIV diagnosis target, most provinces face challenges in reaching the remaining two 90% targets.

## Introduction

Expanded access to HIV testing and antiretroviral treatment (ART) is critical both to reducing levels of AIDS mortality and to reducing HIV incidence, at a population level. This is therefore the focus of the UNAIDS 2020 targets, which aim to achieve a 90% rate of diagnosis in people living with HIV, a 90% rate of ART coverage in HIV-diagnosed individuals and a 90% rate of virological suppression in patients on ART.^1^

However, few African countries have been able to report progress towards these ‘90–90–90’ targets.^2,3^ Most household surveys conducted in African countries do not include questions about whether HIV-positive individuals know they are HIV-positive, which prevents estimation of the fraction of HIV-positive individuals who have been diagnosed. In addition, most African countries have only recently introduced virological monitoring of ART patients, and there is thus limited ability to report on progress towards the last 90% target. This means that the few African studies published to date have relied on special surveys for tracking progress towards the 90–90–90 targets,^4,5^ and almost none have made use of routine monitoring systems.^6^

In South Africa, it has been shown that by triangulating HIV testing data from a number of sources, it is possible to arrive at estimates of the fraction of HIV-positive adults who have been diagnosed positive.^7^ The South African ART programme has also recommended virological monitoring since its inception,^8^ and systems for reporting rates of virological suppression have been established.^9,10^ South Africa is therefore well placed to track its progress towards the 90–90–90 targets. This article aims to estimate progress towards the targets in the period up to 2015, at national and provincial levels.

## Methods

Progress towards the 90–90–90 targets is estimated using the Thembisa model, a combined demographic and HIV model of the South African population. HIV disease progression prior to ART initiation is modelled using a staged model of CD4 decline, with rates of transition between CD4 stages set so that the modelled estimates of the fraction of HIV-positive adults in different CD4 stages match those observed in South African surveys, and HIV mortality assumptions by CD4 stage being set so that the model matches observed trends in mortality by age.^11^

As described previously, the model was fitted to national age-specific HIV prevalence data from antenatal surveys and household surveys to determine key sexual behaviour and HIV transmission parameters.^12^ Separate versions of the model were then created for each of the nine provinces. Key parameters that differed between provinces included the demographic assumptions, marriage rates, initial prevalence of male circumcision, fraction of the population in the ‘high-risk’ and ‘low-risk’ groups, sexual mixing between high- and low-risk groups, initial HIV prevalence and uptake of HIV services (HIV testing, prevention of mother-to-child transmission, ART, medical male circumcision and condoms). To allow for the uncertainty regarding a number of the behavioural parameters, a Bayesian approach was adopted in fitting the model to province-specific HIV prevalence data from antenatal and household surveys.^13^ The model estimates of HIV prevalence were in reasonable agreement with the provincial HIV prevalence data.^13^ The uncertainty regarding the behavioural parameters and the level of HIV prevalence in each province is reflected in the confidence intervals around the model estimates of diagnosis levels and ART coverage.

### Modelling HIV testing

The modelling of HIV testing and diagnosis has been described previously.^7^ Briefly, individuals are assumed to get tested in one of three ways: through antenatal services (women only), through treatment of patients with opportunistic infections (OIs) and through other testing services. The model allows for provincial variation in rates of antenatal HIV testing based on data from the District Health Barometer reports^14,15,16^ and other surveys.^17,18,19^ Proportions of OI patients tested for HIV are assumed to be the same as assumed in the national model, for all provinces, because of lack of province-specific data. Province-specific rates of testing for other reasons are set in such a way that the model estimates of the total number of HIV tests are consistent with estimates of the annual total numbers of HIV tests performed in each province (Online Appendix Figure 4). These province-specific estimates of total HIV tests were derived by disaggregating previously estimated total numbers of HIV tests for the country as a whole.^7^ The totals were calculated for the public health sector, medical schemes, the life insurance industry and other private providers of HIV testing (e.g. workplace HIV testing programmes). Most of the public health sector statistics include provincial disaggregation (from 2004 to 2015), and these were used to calculate the numbers of individuals tested for HIV in the public sector in each province. Information is also available on the provincial profile of HIV testing by insurers^20^ and other private providers.^21^ In the case of medical schemes, data on the provincial profile of individuals tested were not directly available, but rates of HIV testing by province in the Discovery medical scheme^22^ were assumed to apply to other medical schemes in distributing the total HIV tests in medical schemes between provinces. For all three private sector data sources, the fraction of HIV tests in each province that was estimated was assumed to apply in all years, because of the lack of information on temporal changes in provincial distributions. Assumptions about the effect of age, sex and HIV testing history on rates of HIV testing were held constant at the levels estimated previously when the model was fitted to national HIV testing statistics.^7^

### Modelling antiretroviral treatment uptake

The Thembisa model requires as inputs estimates of the total numbers of individuals starting ART in each year, split into three categories (children aged < 15 years, adult males and adult females). These estimates are derived from public sector statistics combined with biennial surveys of numbers of individuals treated in the private and NGO sectors.^23^ Public sector statistics included in the modelling are those from the Comprehensive Care, Management and Treatment (CCMT) reporting system,^24^ in the period prior to 2012, and the District Health Information System (DHIS),^9,25^ in the period from 2012 to 2015. Because of frequent ‘self-transfer’,^26^ many patients who move between ART services are incorrectly recorded as new ART patients, and reporting of ‘new’ ART enrolment is therefore not considered reliable. Instead, annual numbers of new ART patients are modelled using Bayesian B-splines,^27,28^ with the B-splines being fitted to produce estimates of current ART enrolment consistent with reported public and private statistics for each province (Online Appendix Figures 1–3). The model fitting procedure takes into account the change over time in the reporting of ART enrolment (from reporting cumulative enrolment in the period up to 2009 to reporting total current enrolment in subsequent periods, with allowance for provincial differences in the timing of the change in reporting). The model fitting procedure also takes into account possible errors in the reporting (e.g. late reporting and double-counting), with the spline functions ‘smoothing out’ fluctuations because of reporting errors, and with the extent of the fluctuations in the reported totals determining the 95% confidence interval widths. A more detailed statistical description of the B-spline fitting procedure is provided in Online Appendix 1. National ART enrolment was calculated by summing the province-specific totals.

### Modelling viral suppression

Viral suppression is defined in the model as a viral load of less than 400 RNA copies/mL. The model input is the annual rate of viral suppression in patients starting ART with a CD4 count of < 200 cells/µL, and this rate is adjusted to allow for higher rates of viral suppression in patients starting ART at higher CD4 counts.^29^ The input parameters have been estimated from provincial DHIS statistics in 2013/2014, for patients who had been on ART for 48 months (viral load data were available for 55% of these patients).^9^ These rates were 73.4% in Eastern Cape, 80.1% in Free State, 72.5% in Gauteng, 84.2% in KwaZulu-Natal, 67.5% in Limpopo, 68.2% in Mpumalanga, 84.9% in North West, 75.8% in Northern Cape and 84.8% in Western Cape. Because of lack of historical data on viral suppression, the same input parameter was assumed to apply in all years.

The Thembisa model is programmed in C++, and all results presented are based on the C++ version of the model. An Excel version of the model, as well as outputs from the Excel model, is available for download from the Thembisa website (www.thembisa.org).

## Results

**FIGURE 1:**
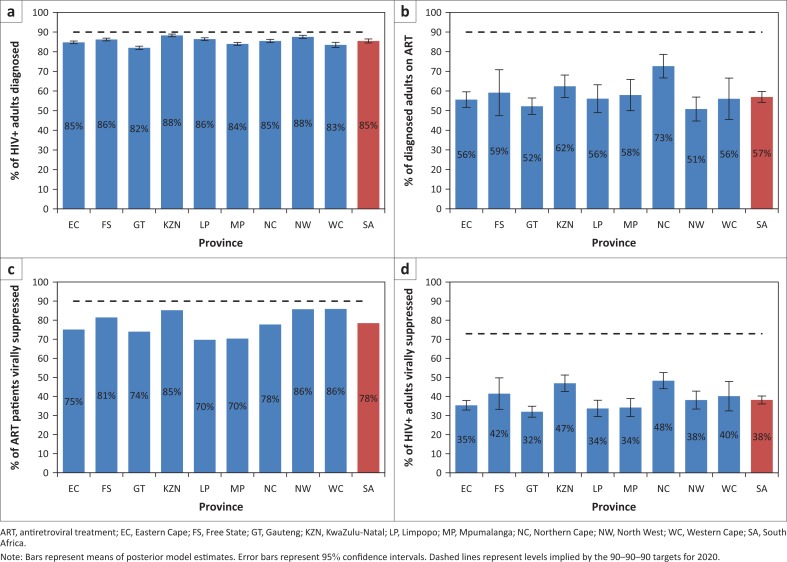
Provincial progress towards the 90–90–90 targets in 2015: (a) Proportion of HIV-positive adults diagnosed; (b) Proportion of diagnosed adults on ART; (c) Proportion of ART patients virally suppressed; (d) Proportion of HIV-positive adults on ART and virally suppressed.

By the middle of 2015, high levels of HIV diagnosis were achieved in South Africa, with an estimated 85.5% (95% CI: 84.5% – 86.5%) of HIV-positive adults diagnosed. Rates of HIV diagnosis were similar across provinces, ranging from 82.0% in Gauteng to 88.3% in KwaZulu-Natal (Figure 1a).

Table 1 shows that 3.39 million (95% CI: 3.26–3.52 million) South Africans were on ART by mid-2015, a more than 30-fold increase on the total in 2005 (103 300, 95% CI: 100 900–105 600). Approximately 287 000 ART patients in 2015 (8.5%) were receiving treatment from the private sector or NGOs. Over the period from mid-2010 to mid-2013, the annual number of new ART patients was relatively stable at around 560 000 per annum, but in the more recent years enrolment declined, reaching 413 000 (95% CI: 342 000–486 000) over the period from mid-2014 to mid-2015 (Table 2). The decline in annual new enrolment was particularly pronounced in children: from 39 500 (95% CI: 36 000–43 100) in 2010–2011 to 13 700 (95% CI: 9600–18 900) in 2014–2015.

**TABLE 1:** Numbers of patients currently on antiretroviral treatment in South Africa.

Category and variable	2004	2005	2006	2007	2008	2009	2010	2011	2012	2013	2014	2015
**By sex/age**												
Men	17 700	34 900	63 000	113 000	178 000	265 000	383 000	533 000	681 000	837 000	969 000	1 081 000
Women	24 400	59 200	117 000	218 000	350 000	522 000	745 000	1 044 000	1 349 000	1 651 000	1 908 000	2 134 000
Children (<15)	3400	9100	18 000	35 000	55 000	82 000	110 000	140 000	160 000	173 000	177 000	174 000
**By province**												
Eastern Cape (EC)	5200	11 600	22 000	38 000	61 000	92 000	132 000	186 000	237 000	287 000	327 000	359 000
Free State (FS)	2300	4000	7000	14 000	25 000	42 000	64 000	91 000	119 000	147 000	169 000	187 000
Gauteng (GT)	12 400	29 200	54 000	95 000	144 000	208 000	292 000	409 000	521 000	631 000	712 000	774 000
KwaZulu-Natal (KZ)	13 500	27 500	54 000	106 000	173 000	262 000	376 000	521 000	665 000	807 000	933 000	1 045 000
Limpopo (LP)	2100	4400	9000	19 000	34 000	54 000	81 000	117 000	148 000	176 000	198 000	216 000
Mpumalanga (MP)	3300	5800	11 000	23 000	37 000	57 000	84 000	121 000	165 000	217 000	267 000	316 000
Northern Cape (NC)	400	1200	3000	6000	9000	12 000	15 000	19 000	24 000	31 000	38 000	46 000
North West (NW)	3000	7800	16 000	28 000	46 000	63 000	89 000	122 000	149 000	173 000	191 000	204 000
Western Cape (WC)	2500	10 100	20 000	32 000	47 000	65 000	85 000	108 000	131 000	154 000	173 000	190 000

**Total**	**45 500**	**103 300**	**199 000**	**366 000**	**583 000**	**869 000**	**1 238 000**	**1 717 000**	**2 190 000**	**2 661 000**	**3 054 000**	**3 389 000**

ART, antiretroviral treatment.

Note: All numbers are rounded to the nearest 1000 (except for ART totals in 2004–2005, which are rounded to the nearest 100), and are estimated at the middle of each year.

**TABLE 2:** Numbers of patients starting antiretroviral treatment in South Africa.

Category and variable	2003/2004	2004/2005	2005/2006	2006/2007	2007/2008	2008/2009	2009/2010	2010/2011	2011/2012	2012/2013	2013/2014	2014/2015
**By sex/age**												
Men	10 500	21 400	36 000	61 000	79 000	106 000	143 000	179 000	178 000	188 000	161 000	141 000
Women	14 500	41 500	70 000	118 000	154 000	200 000	256 000	342 000	348 000	346 000	296 000	259 000
Children (<15)	2000	6200	10 200	17 800	23 300	30 900	34 600	39 500	31 900	26 400	18 500	13 700

**Total**	**26 900**	**69 100**	**115 000**	**197 000**	**257 000**	**337 000**	**433 000**	**560 000**	**558 000**	**559 000**	**475 000**	**413 000**

ART, antiretroviral treatment.

Note: All numbers are rounded to the nearest 1000 (except for new paediatric ART enrolment and new adult enrolment in 2003–2005, which are rounded to the nearest 100). Totals represent new enrolment over the period from midyear to midyear.

Expressed as a fraction of all HIV-positive individuals, ART coverage in South Africa in 2015 was 48.6% (95% CI: 46.0% – 51.2%), more than double the ART coverage in 2010 (Table 3). ART coverage was substantially higher in women (52.0%, 95% CI: 49.3% – 54.7%) than in men (43.2%, 95% CI: 40.2% – 46.2%), with coverage in children being between the two (47.4%, 95% CI: 44.0% – 50.8%). Coverage differed substantially by province, ranging from 43.0% (95% CI: 40.9% – 45.2%) in Gauteng to 62.0% (95% CI: 58.4% – 64.9%) in Northern Cape. Similar differences were observed when coverage was expressed as a fraction of all HIV-diagnosed adults: 56.9% (95% CI: 55.3% – 58.7%) of all HIV-diagnosed adults were on ART, with this proportion varying between 50.8% (95% CI: 47.5% – 54.6%) in North West and 72.7% (95% CI: 68.8% – 75.8%) in Northern Cape (Figure 1b).

**TABLE 3:** Antiretroviral treatment coverage (as a fraction of all HIV-positive individuals).

Category and variable	2004		2005		2006		2007		2008		2009		2010		2011		2012		2013		2014		2015	
											
	%	95% CI	%	95% CI	%	95% CI	%	95% CI	%	95% CI	%	95% CI	%	95% CI	%	95% CI	%	95% CI	%	95% CI	%	95% CI	%	95% CI
**By sex/age**																								
Men	1.0	0.9–1.1	1.9	1.8–2.0	3.3	3.1–3.6	5.7	5.3–6.1	8.6	8.1–9.2	12.4	11.6–13.2	17.3	16.2–18.4	23.2	21.7–24.7	28.9	27.0–30.8	34.7	32.4–37.0	39.3	36.7–42.0	43.2	40.2–46.2
Women	1.0	0.9–1.0	2.2	2.1–2.3	4.2	4.0–4.3	7.3	7.0–7.7	11.2	10.8–11.7	15.9	15.3–16.5	21.7	20.8–22.5	29.2	27.9–30.4	36.2	34.7–37.8	42.9	40.9–44.8	48.0	45.7–50.2	52.0	49.3–54.7
Children	0.9	0.9–1.0	2.4	2.2–2.5	4.5	4.3–4.8	8.2	7.7–8.6	12.7	12.0–13.3	18.6	17.7–19.6	25.3	24.1–26.5	32.9	31.4–34.5	38.8	36.9–40.7	43.4	41.0–45.7	46.0	43.2–48.9	47.4	44.0–50.8
**By province**																								
Eastern Cape	1.1	1.0–1.1	2.2	2.1–2.3	3.9	3.8–4.0	6.6	6.4–6.7	10.0	9.8–10.2	14.2	14.0–14.5	19.6	19.3–20.0	26.6	26.0–27.2	33.0	32.3–33.7	39.0	38.0–40.1	43.5	42.2–44.8	46.9	45.2–48.6
Free State	0.7	0.7–0.8	1.2	1.2–1.3	2.2	2.1–2.3	4.1	3.9–4.3	7.2	6.9–7.5	12.1	11.6–12.6	18.2	17.4–19.0	25.7	24.5–27.0	33.2	31.2–35.2	40.8	37.9–43.7	46.6	43.1–50.0	51.1	46.0–56.3
Gauteng	1.2	1.1–1.3	2.6	2.4–2.8	4.6	4.3–4.9	7.5	7.0–8.0	10.6	10.0–11.3	14.4	13.6–15.3	19.3	18.3–20.4	25.9	24.4–27.4	31.8	29.9–33.7	37.2	34.7–39.8	40.7	37.7–43.7	43.0	39.4–46.5
KwaZulu-Natal	0.9	0.9–1.0	1.8	1.7–1.9	3.4	3.3–3.6	6.5	6.2–6.8	10.3	9.9–10.8	15.3	14.7–16.0	21.5	20.4–22.6	29.1	27.0–31.2	36.4	33.6–39.2	43.5	39.9–47.1	49.6	45.5–53.7	54.9	49.8–60.0
Limpopo	0.8	0.7–0.8	1.5	1.4–1.6	3.0	2.8–3.1	5.9	5.5–6.3	9.8	9.3–10.3	14.8	14.1–15.5	21.0	19.9–22.0	28.6	26.7–30.6	35.1	32.3–37.8	40.7	37.0–44.3	44.8	39.9–49.7	47.8	41.7–53.9
Mpumalanga	0.7	0.7–0.8	1.2	1.1–1.3	2.2	2.0–2.3	4.1	3.8–4.4	6.6	6.2–7.0	10.0	9.3–10.6	14.4	13.4–15.4	20.3	18.5–22.2	27.1	24.3–29.8	34.7	30.8–38.6	41.7	36.8–46.5	48.2	41.6–54.9
Northern Cape	0.9	0.8–1.0	2.3	2.1–2.5	4.9	4.5–5.2	9.7	8.8–10.6	13.4	12.4–14.3	16.8	15.8–17.8	21.1	19.8–22.3	26.4	24.8–28.0	33.3	31.2–35.4	41.9	38.7–45.1	51.4	47.2–55.5	62.0	56.7–67.3
North West	0.7	0.7–0.8	1.9	1.7–2.1	3.9	3.5–4.2	7.2	6.6–7.9	11.4	10.5–12.4	16.0	14.7–17.3	21.7	19.9–23.4	28.3	25.9–30.7	33.9	30.8–36.9	38.7	34.6–42.8	41.9	37.2–46.7	44.2	38.8–49.6
Western Cape	1.3	1.2–1.4	4.7	4.3–5.0	8.3	7.8–8.8	12.3	11.6–13.0	16.6	15.8–17.4	21.4	20.4–22.4	26.4	25.2–27.6	31.8	30.1–33.5	36.6	34.4–38.7	40.9	38.0–43.7	44.2	40.4–48.0	46.9	42.3–51.5

**Total**	**1.0**	**0.9–1.0**	**2.1**	**2.0–2.2**	**3.9**	**3.7–4.0**	**6.8**	**6.5–7.1**	**10.4**	**10.0–10.7**	**14.8**	**14.3–15.4**	**20.3**	**19.6–21.1**	**27.3**	**26.1–28.4**	**33.7**	**32.3–35.2**	**39.9**	**38.1–41.7**	**44.7**	**42.6–46.9**	**48.6**	**46.0–51.2**

CI, confidence intervals.

The fraction of ART patients who were virologically suppressed was 78.4% nationally (Figure 1c). Rates of virological suppression differed substantially between provinces, ranging from 69.7% in Limpopo and 70.3% in Mpumalanga to 85.8% in North West and 85.9% in Western Cape. Overall, the fraction of HIV-positive adults who were on ART and virologically suppressed in 2015 was 38.2% (95% CI: 36.7% – 39.7%); the proportion varied from 31.8% (95% CI: 29.2%–34.4%) in Gauteng to 48.3% (95% CI: 45.4% – 50.7%) in Northern Cape (Figure 1d).

**FIGURE 2:**
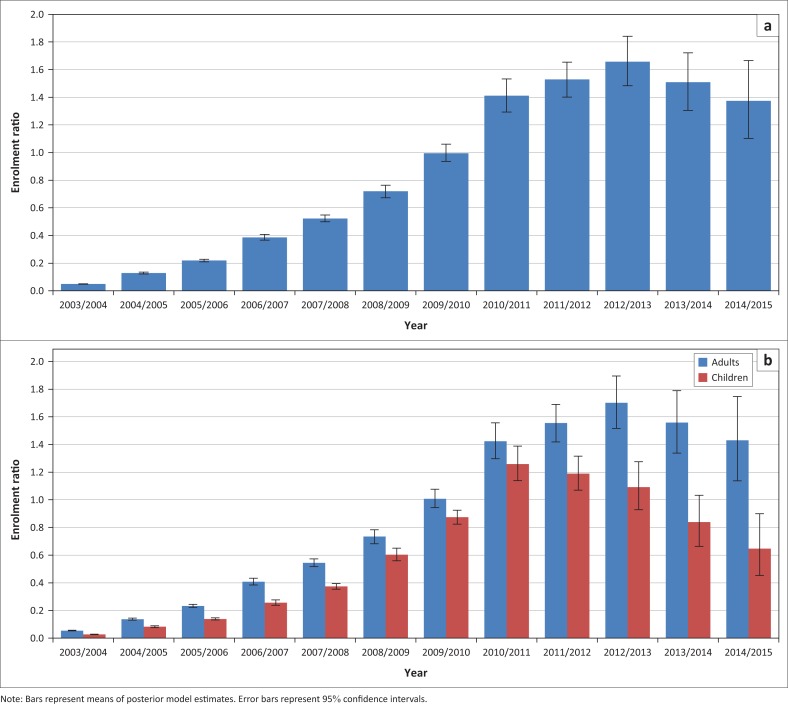
ART enrolment ratio (new ART initiation divided by new HIV infections): (a) National average; (b) Comparison of adults and children.

Expressed as a proportion of new HIV infections, new ART enrolment rose to 1.00 (95% CI: 0.94–1.06) in the 2009–2010 period, then continued to increase in the subsequent years as ART eligibility criteria were revised and as HIV incidence declined (Figure 2a). However, the enrolment ratio dropped from 1.66 (95% CI: 1.48–1.84) in 2012–2013 to 1.37 (95% CI: 1.10–1.67) in 2014–2015. Although the drop was not significant in adults, the enrolment ratio declined significantly in children, from 1.26 (95% CI: 1.14–1.39) in 2010–2011 to 0.65 (95% CI: 0.45–0.90) in 2014–2015 (Figure 2b).

## Discussion

This study shows that South Africa has made good progress towards the first UNAIDS 90% target, with progress being relatively uniform across provinces. Progress towards the 90% diagnosis target is similar to that made in Botswana^4^ and the Chiradzulu district in Malawi^5^ (Table 4). However, progress towards the second and third UNAIDS 90% targets has been less impressive, with much variation between provinces. Despite its success in getting HIV-positive individuals diagnosed, South Africa has not achieved the high levels of ART coverage and viral suppression seen in Botswana, Rwanda and Malawi (Table 4).

**TABLE 4:** Comparison with 90–90–90 estimates from other studies.

Study	Location	Year	Ages	% of HIV+ diagnosed	% of HIV+ on ART	% of HIV+ on ART and suppressed	Suppression definition
**Other African countries**							
Gaolathe et al.^4^	Botswana	2013–2015	16–64	87.0	72.8	70.2	VL < 400
Maman et al.^5^	Chiradzulu, Malawi	2013	15–59	77.0	62.5	56.9	VL < 1000
Nsanzimana et al.^6^	Rwanda	2013	All	-	71.0	52	VL < 40
**South African studies**							
Lippman et al.^47^	Lekwe-Teemane, Greater Taung (NW)	2014	18–49	65.8	46.1	-	
Huerga et al.^31^	Mbongolwane, Eshowe (KZ)	2013	15-59	75.2	53.1†	49.4†	VL < 1000
Iwuji et al.^35^	UMkhanyakude (KZ)	2012–2014	16+	78.8	38.2	-	
Jean et al.^48^	Orange Farm (GT)	2012	18+	-	27.2†	25.0†	VL < 400
Van Rooyen et al.^49^	Vulindlela (KZ)	2011–2012	18+	63.7	31.8	-	
**Thembisa model estimates**							
South Africa	-	2015	15+	85.5	48.7	38.2	VL < 400
South Africa	-	2014	15+	83.1	44.7	34.9	VL < 400
South Africa	-	2013	15+	80.8	39.7	31.0	VL < 400
South Africa	-	2012	15+	76.7	33.4	26.0	VL < 400
South Africa	-	2011	15+	71.1	26.8	20.8	VL < 400
**UNAIDS targets**	-	2020	All	90.0	81.0	72.9	VL < 1000

VL, viral load; ART, antiretroviral treatment.

† ,Based on presence of antiretroviral drugs in blood specimens; estimates of viral suppression exclude poorly adherent individuals who do not have antiretroviral drugs detectable in their blood.

These results are similar to the results of other South African surveys. The model estimate of the ART coverage in 2012 (33.7%, 95% CI: 32.3% – 35.2%) is consistent with the results of a national survey in the same year (31.2%, 95% CI: 28.1% – 34.5%).^30^ The model estimates that 26.0% of all HIV-positive adults were on ART and virally suppressed in 2012, which is consistent with an estimate of 24% based on estimates from the national laboratory system,^10^ as well as a smaller survey in Gauteng (Table 4). Estimates of ART coverage in other local household surveys are also consistent with model estimates (Table 4), although a survey conducted in Mbongolwane and Eshowe districts found higher ART coverage and viral suppression than expected, probably because special HIV interventions were introduced in these districts prior to the survey.^31^

Provincial differences in the scale-up of HIV testing and ART access may be partly explained by differences in budget allocations. In a recent analysis of HIV expenditure by province, it was found that the HIV expenditure per HIV-positive individual was highest in Northern Cape and Free State, and lowest in Gauteng and Mpumalanga.^32^ This could explain why progress towards the 90–90–90 targets is greatest in Northern Cape, whereas progress appears relatively poor in Gauteng and Mpumalanga (Figure 1). It is important that the underfunding of the HIV response in the latter group of provinces is corrected.

The slowdown in adult ART enrolment in recent years might be considered surprising, given the broadening of ART eligibility criteria in August 2011^33^ and January 2015.^34^ Poor linkage to care following diagnosis is likely to be a key explanation; in a recent randomised trial in rural KwaZulu-Natal, less than half of HIV-positive adults not in care sought HIV care within six months.^35^ Even when linked to HIV care, adults with higher CD4 counts have significantly lower rates of ART initiation,^36,37,38,39,40,41^ and thus the broadening of ART eligibility criteria to include patients in higher CD4 categories may have less impact on enrolment than might be expected if patients at higher CD4 counts had the same rates of ART initiation as patients at lower CD4 counts. Simplified models for ART initiation may be required to increase the fraction of HIV-diagnosed adults on ART.^42,43^ In addition, further research is required to better understand the barriers to ART initiation in HIV-diagnosed individuals, particularly those at higher CD4 counts. Efforts to improve the transition from diagnosis to ART initiation should also focus particularly on poorly performing provinces such as North West and Gauteng (Figure 1b).

It is concerning that annual new ART enrolment in children has declined so substantially in the last five years. Although this is partly because of the success of prevention of mother-to-child transmission programmes, new enrolment has declined even when expressed as a fraction of annual new infections (Figure 2b). This might be because of inadequate HIV testing: Although great emphasis has been placed on HIV testing in early infancy,^44^ there has been little focus on HIV testing in children after infancy and in children who are not known to have been exposed to HIV. It is likely that an increasingly high fraction of mother-to-child transmission is postnatal transmission and transmission from mothers who have not been diagnosed positive, and thus an increasingly high fraction of transmission is likely to be missed by the current screening strategy. In addition, national HIV testing statistics and targets until recently excluded testing under the age of 15,^45,46^ and the absence of any monitoring of HIV testing in the 1–14 year age group means that it has not been possible to produce estimates of the fraction of HIV-positive children who have been diagnosed. A limitation of this study is therefore that it does not assess progress towards the 90–90–90 targets in children – a limitation common to most studies (Table 4). It is important that children are not neglected in the scale-up of HIV testing and ART,^50^ and there is an urgent need for better monitoring of HIV testing and diagnosis in children.

Viral suppression has been identified as the most important determinant of future HIV incidence trends in South Africa,^12^ and it is therefore concerning that rates of viral suppression are as low as 70% in Limpopo and Mpumalanga. Efforts to improve viral suppression could include adherence support interventions,^51^ community-supported models of care to improve retention,^52^ better supply chain management to avoid drug stock-outs and potentially new drugs, such as dolutegravir.^53^ Efforts are also required to monitor viral suppression more thoroughly, as the data on which these model estimates are based represent only 55% of adults starting ART in 2009–2010 who were followed up in 2013–2014 (the fraction of patients who had viral load results varied between 42% in Limpopo and 65% in Eastern Cape). Although viral suppression statistics are also available at other ART durations, these are generally similar to the rates at 48 months, and are based on less complete information.^9^ A limitation of this analysis is that it does not quantify the uncertainty because of the incomplete viral load data, but it is anticipated that it will be possible to produce confidence intervals for the modelled rate of viral suppression in future, as more data become available. To be consistent with the published statistics, we have used a viral load threshold of 400 copies/mL in defining suppression, although guidelines issued by the WHO^54^ and Global AIDS Response Progress Reporting^55^ recommend using a threshold of 1000 copies/mL. The Thembisa model estimates that using a threshold of 1000 copies/mL would increase the rate of viral suppression in 2015 from 78.4% to 81.7%.

Another limitation is that the confidence intervals around the provincial ART coverage estimates are too wide to draw firm conclusions about the relative performance of the different provinces. The wide confidence intervals are mainly because of erratic and infrequent reporting of ART totals in recent years (see Online Appendix 1). Further work is required to correct anomalies in the DHIS data and to integrate more frequent DHIS estimates into the Thembisa model fitting procedure, which should lead to narrower confidence intervals. An additional limitation is that the ART enrolment ratio that we have proposed^56^ may become meaningless in future if the annual number of new infections (the denominator in the calculation) declines towards zero. A strength of this analysis is that it employs a fully integrated HIV transmission and survival model, unlike previous analyses of ART coverage in South Africa, which have relied on independent models to estimate HIV incidence and HIV survival.^57,58^ This study estimates slightly lower levels of ART uptake than estimated previously (e.g. 1.72 million on ART in 2011 compared to 1.79 million [95% CI: 1.65–1.93 million]^57^) because the earlier study assumed an immediate transition from reporting cumulative totals to reporting current totals in 2009, when in fact the transition occurred more gradually in some provinces.

Achieving the 90–90–90 targets will require that at least 73% of HIV-positive individuals are on ART and virally suppressed by 2020. With the proportion treated and suppressed at 38% in 2015, South Africa still has a long way to go towards meeting the targets. However, the successes seen in Botswana, Rwanda and Malawi offer hope that the targets can be achieved, and South Africa needs to learn from these success stories if it is to maximise the impact of its ART programme.
